# Amino acid–derived quorum sensing molecules controlling the virulence of vibrios (and beyond)

**DOI:** 10.1371/journal.ppat.1007815

**Published:** 2019-07-11

**Authors:** Tom Defoirdt

**Affiliations:** Center for Microbial Ecology and Technology (cmet), Ghent University, Gent, Belgium; Duke University School of Medicine, UNITED STATES

## Quorum sensing systems as targets for the development of novel drugs to control bacterial disease

Antibiotic-resistant bacteria are currently posing a major threat to our society, rendering several human and animal infections hard to cure, and this situation is predicted to become even more precarious in the near future if no adequate measures are undertaken [1]. Bacterial pathogens synthesize different compounds and structures that enable them to colonize and damage their host—i.e., virulence factors. As virulence factors are required for infection, preventing pathogens from producing them constitutes an important alternative strategy for the control of bacterial diseases: antivirulence therapy [2]. One of the most intensively studied targets in this respect is quorum sensing, bacterial cell-to-cell communication in which bacteria coordinate the expression of certain genes in response to the presence of small molecules. In the past years, several criteria have been proposed to define the quorum sensing phenomenon and the small molecules involved in it (e.g., discussed in [3–6]). In the current manuscript, I will use the generic term “quorum sensing molecule” to refer to a small molecule produced by a bacterium under certain conditions that generates a phenotypic response that extends beyond changes required to metabolize or detoxify the molecule. Various quorum sensing molecules have been documented to control the production of virulence factors in many bacterial pathogens of plants, animals, and humans (for reviews, see [7–10]).

Vibrios (Gammaproteobacteria belonging to the genus *Vibrio*) are gram-negative, usually motile rods that are ubiquitous in aquatic environments, and several strains are major pathogens of aquatic organisms and humans, causing major environmental, economic, and public health impacts [11]. These impacts will likely increase given the increased geographical spread of vibrios as a result of climate change [12]. Moreover, like other bacterial pathogens, vibrios are acquiring antibiotic resistance, thereby rendering the currently used antibiotic treatments ineffective [13]. Hence, it should be no surprise that vibrios are among the most intensively studied model organisms in quorum sensing studies. In fact, the quorum sensing principle was originally discovered in *Vibrio fischeri* [14]. Many other gram-negative bacteria (including other *Vibrio* species such as *V*. *anguillarum*) contain a similar quorum sensing system based on the production and detection of acylated homoserine lactone (AHL) quorum sensing molecules. These systems consist of a homolog of the *V*. *fischeri* LuxI AHL synthase enzyme and a homolog of the *V*. *fischeri* LuxR transcriptional regulator that detects the AHL and subsequently binds to the promoter of the quorum sensing target genes and thereby affects expression of these genes.

In addition to the relatively simple AHL systems, vibrios also contain complex multichannel quorum sensing systems [8,15,16]. These systems rely on the production and detection of multiple signal molecules with different chemical structures that are detected by dedicated membrane-bound receptors that feed a shared signal transduction cascade [17]. During the past decades, various inhibitors of AHL and multichannel quorum sensing systems have been reported (for recent reviews, see [9,10]). Although interfering with these quorum sensing systems shows promise to control disease caused by some *Vibrio* species (e.g., Harveyi clade vibrios and *V*. *cholerae* [10,18]), it has no impact on the virulence of other species (e.g., *V*. *anguillarum* [15,19]). Therefore, it is important to identify additional targets for antivirulence therapy. Interestingly, more quorum sensing molecules are still being discovered, thereby increasing the possibilities for the development of novel virulence-inhibitory therapies. Here, I will focus on three quorum sensing molecules that were recently documented to control the virulence of vibrios: indole, cyclo(L-phenylalanine-L-proline) (cyclo(Phe-Pro)), and 3,5-dimethylpirazin-2-ol (DPO). These quorum sensing molecules share one particular feature—i.e., they are derived from amino acids.

### Impact of indole on the virulence of bacterial pathogens

**Fig 1:**
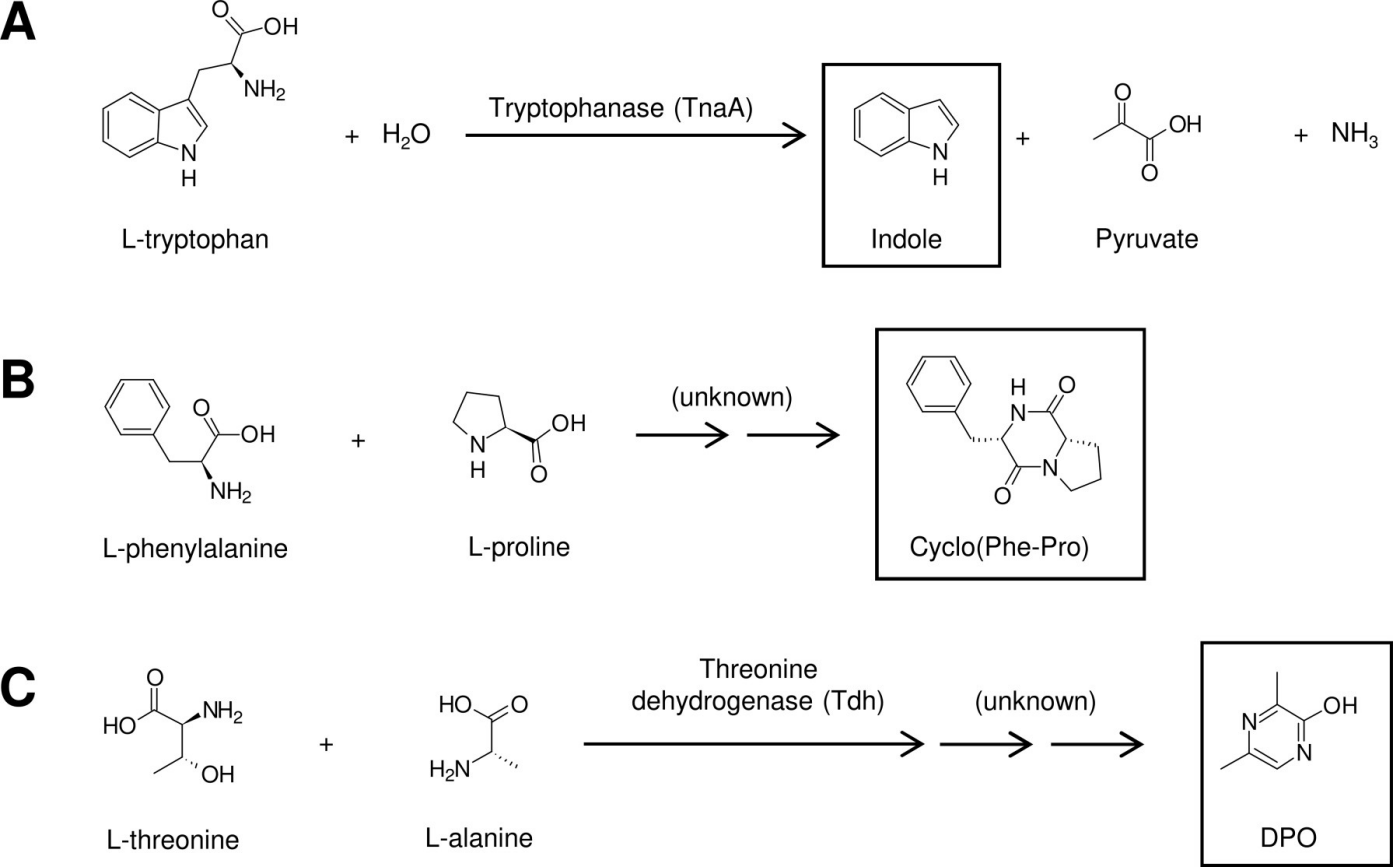
The amino acid–derived quorum sensing molecules indole (A), cyclo(Phe-Pro) (B), and DPO (C) and enzymes involved in their biosynthesis. Pathways that have not been fully characterized are also indicated (unknown). cyclo(Phe-Pro), cyclo(L-phenylalanine-L-proline); DPO, 3,5-dimethylpirazin-2-ol.

Although different bacteria have been known for quite some time to produce indole from tryptophan during stationary phase (**Fig 1A**), the appreciation of indole as a quorum sensing molecule is of relatively recent origin. The compound controls various virulence-related phenotypes (most notably, biofilm formation and motility) in human, animal, and plant pathogens [20].

Indole affects biofilm formation in *V*. *anguillarum*, *V*. *campbellii*, and *V*. *cholerae*, and this was linked to the production of exopolysaccharides (which are a major constituent of the extracellular matrix in biofilms). In *V*. *cholerae*, biofilm levels increased in the presence of higher indole concentrations [21], whereas in the other two species, the opposite effect was observed [22,23]. This might reflect differences in the lifestyles of these bacteria. Indeed, when compared with marine vibrios, various regulatory responses have been reported to be opposite in *V*. *cholerae* (e.g., the down-regulation of virulence factors and biofilm formation by the multichannel quorum sensing system [15]). In fact, an impact of indole on biofilm formation seems to be a general feature that is not limited to vibrios, and other bacteria (such as *Acinetobacter oleivorans*, *Agrobacterium tumefaciens*, *Escherichia coli*, and *Pseudomonas aeruginosa*) also showed altered biofilm formation (either increased or decreased) in the presence of indole [24–27]. In addition to affecting biofilm formation, indole also decreased the (expression of genes involved in) flagellar motility of *V*. *campbellii* and *V*. *cholerae* [21,23], which is also consistent with observations in other bacteria such as *E*. *coli* [28], *Salmonella enterica* serovar Typhimurium [29], and *P*. *aeruginosa* [25]. Apart from biofilm formation and motility, other virulence factors have been reported to be affected by indole in specific *Vibrio* species as well (**Table 1**), and most importantly, the addition of indole decreased the virulence of vibrios in invertebrate and vertebrate host infection models [22,23]. Finally, many other vibrios, including (but not limited to) *V*. *mediterranei*, *V*. *nigripulchritudo*, *V*. *orientalis*, and *V*. *parahaemolyticus*, are known to produce indole [20]. However, a phenotypic response to indole has not yet been documented in these species.

**Table 1:** The impact of the amino acid–derived quorum sensing molecules indole, cyclo(Phe-Pro), and DPO on virulence-related phenotypes in vibrios.

Molecule	Species	Phenotypes affected	References
Indole	*V*. *anguillarum*	Biofilm ↓, exopolysaccharide ↓, virulence (sea bass) ↓	[22]
	*V*. *campbellii*	Biofilm ↓, exopolysaccharide ↓, motility ↓, quorum sensing ↓, virulence (brine shrimp and giant river prawn) ↓	[23]
	*V*. *cholerae*	Biofilm ↑, grazing resistance ↑, cell envelope production and maintenance ↑^1^, exopolysaccharide (VPS) ↑^1^, motility ↓^1^	[21]
	*V*. *splendidus*	Protease, hemolysin and ABC transporter ATP-binding protein ↓^1^	[30]
Cyclo(Phe-Pro)	*V*. *cholerae*	Cholera toxin and coregulated pilus ↓	[31,32]
	*V*. *vulnificus*	Outer membrane protein OmpU ↑^1^, biofilm ↓, resistance to oxidative stress ↑	[33–35]
DPO	*V*. *cholerae*	Biofilm ↓, exopolysaccharide (VPS) ↓^1^, Rtx toxin ↓	[36,37]

^1^ Effect on gene expression level based on promoter activity, microarray, and/or RT qPCR studies; impact on phenotypes not documented.

Abbreviations: ABC, ATP binding cassette; cyclo(Phe-Pro), cyclo(L-phenylalanine-L-proline); DPO, 3,5-dimethylpirazin-2-ol, OmpU, outer membrane protein U; RT qPCR, reverse transcriptase quantitative PCR; Rtx, repeats in toxin; VPS, *Vibrio* polysaccharide

Despite the fact that indole has a quorum sensing function in various species, thus far, an indole receptor has not been definitively identified for any bacterium, although some candidates have been proposed (e.g., the transcriptional regulator SdiA in *E*. *coli*) [38]. However, several connections with other components of the virulence regulatory network have been described. One common theme seems to be that indole signaling and the stress sigma factor RpoS are interconnected [21–23]. Furthermore, in *V*. *campbellii*, indole decreases the activity of the multichannel quorum sensing system [23], whereas no such effect was observed in *V*. *cholerae* [21]. Indole has also been reported to interfere with AHL-based quorum sensing in a number of gram-negative bacteria (including *A*. *oleivorans*, *Chromobacterium violaceum*, *P*. *aeruginosa*, *P*. *chlororaphis*, and *Serratia marcescens*)—probably by interfering with the stability and folding of LuxR-type AHL receptors [26,39].

## cyclo(phe-pro) as virulence-regulating quorum sensing molecule

Cyclic dipeptides (or 2,5-diketopiperazines) are bioactive molecules produced through the combination of two amino acids by nonribosomal peptide synthetases or cyclodipeptide synthases in a wide range of organisms [40]. Vibrios belonging to the species *V*. *cholerae*, *V*. *harveyi*, *V*. *parahaemolyticus*, and *V*. *vulnificus* produce the cyclic dipeptide cyclo(Phe-Pro) (**Fig 1B**), with a maximal level being produced at the onset of stationary phase [33]. Although enzymes from the nonribosomal peptide synthetase and cyclopeptide synthase families are the major catalysts known for cyclic dipeptide formation, the exact biosynthetic route of cyclo(Phe-Pro) in vibrios is yet unknown. Two decades ago, cyclic dipeptides were documented to activate AHL reporter strains (although at higher concentrations than AHLs) [41]. However, the actual biological function of these compounds in the producing species remained unknown until, more recently, cyclo(Phe-Pro) was reported to induce the production of the major outer membrane protein U (OmpU), a virulence factor in various vibrios [33], whereas it inhibited cholera toxin and toxin-coregulated pilus production in *V*. *cholerae* [31]. Furthermore, cyclo(Phe-Pro) decreased biofilm formation of *V*. *vulnificus* in a concentration-dependent way [34]. Very recently, cyclo(Phe-Pro) was reported to increase the resistance of *V*. *cholerae*, *V*. *parahemolyticus*, and *V*. *vulnificus* to reactive oxygen (one of the reactions of the innate immune defense in higher organisms) by activating the expression of catalase-peroxidase (*katG*) [35].

The response to cyclo(Phe-Pro) requires the membrane-bound regulator ToxR, a major virulence regulator in vibrios [32,33]. The mechanism by which cyclo(Phe-Pro) affects ToxR is not yet clear, although the periplasmic domain of ToxR and the LysR family regulator LeuO are required for the activity [32,35]. Furthermore, the cyclo(Phe-Pro)–ToxR–LeuO pathway also represses *aphA*, one of the master regulators of the multichannel quorum sensing system in *V*. *cholerae* [32]. Finally, cyclo(Phe-Pro) is also connected with the RpoS regulon in *V*. *cholerae*, *V*. *parahemolyticus*, and *V*. *vulnificus* because the *rpoS* mRNA was stabilized in the presence of exogenous cyclo(Phe-Pro) [35].

## The most recently discovered player: DPO

Very recently, DPO has been identified as a quorum sensing molecule in *V*. *cholerae* [37]. Although the biosynthetic pathway has not yet been completely elucidated, DPO was found to be derived from L-threonine and L-alanine (**Fig 1C**). The threonine dehydrogenase (*tdh*) gene is required for DPO biosynthesis, and the molecule is detected by the cytoplasmic transcription factor VqmA [36]. Upon binding of DPO, VqmA activates transcription of the small RNA *vqmR*, which in turn represses the repeats in toxin (*rtx*) toxin genes and the *vpsT* transcriptional regulator of biofilm formation [36]. The addition of exogenous DPO (100 μM) decreased the expression of the *Vibrio* polysaccharide (*vps*) genes and inhibited biofilm formation in *V*. *cholerae* [37]. The impact on other virulence-related phenotypes has not yet been documented.

The fact that threonine dehydrogenases are conserved among bacteria, together with the observation that cell-free supernatants of *E*. *coli* also contain DPO activity [37], suggests that DPO might be produced by a wide variety of bacteria. However, it is less clear whether non-*Vibrio* species are also able to respond to DPO because homologs of the VqmA receptor are limited to the genus *Vibrio*. Furthermore, the *vqmR* gene is highly conserved among the Vibrionaceae [36], suggesting that other vibrios in addition to *V*. *cholerae* might be able to respond to DPO. Remarkably, a functional *vqmA* gene was very recently identified in the genome of a vibriophage. The phage-encoded VqmA controls genes required for lysis, resulting in lysis of phage-infected *Vibrio* cells in the presence of DPO [42]. These observations further broaden the scope of influence of quorum sensing molecules with respect to interactions between bacteria, viruses, and eukaryotes.

## Ecological perspective in the context of the life cycle of pathogenic vibrios

The life cycle of pathogenic vibrios is characterized by an alteration of a host-associated and an external phase in the aquatic environment, and each of these transitions requires major metabolic changes [43]. During passage through the gastrointestinal tract of a host, bacteria experience a shift in fermentation pattern, with easily degradable carbohydrates being metabolized in the proximal part and more refractory compounds (including proteins) in the distal part [44]. Amino acid–derived quorum sensing molecules could enable vibrios to sense the shift to protein fermentation toward the distal part of the gastrointestinal tract, thereby facilitating the metabolic changes that are required to survive in the external environment. This notion is further supported by a number of observations, including the interconnection between the production of and response to amino acid–derived quorum sensing molecules and the stress sigma factor RpoS (which has been demonstrated for indole and cyclo(Phe-Pro) [23,35], whereas it has not been investigated yet for DPO), the fact that these molecules are all mainly produced during (early) stationary phase and are active within the same concentration range (roughly 0.1–1 mM) [20,33,37], and transcriptomic analyses showing that sensing of these molecules leads to alterations in genes involved in energy production and amino acid transport and metabolism [23,34].

Because they are derived from different amino acids, it is tempting to speculate that the bacteria might be able to sense and respond to the amino acid landscape in their surroundings. Several vibrios are able to associate with and/or infect various host organisms [45], and distinguishing the amino acid composition of their surroundings (which likely will be different in different hosts) might enable the regulatory network of these bacteria to adapt the production of virulence factors to the specific host they encounter. In order to clarify the previously mentioned hypotheses, further research is needed with respect to the biosynthetic and response pathways of these quorum sensing molecules, the relation with the amino acid composition of the environment, and possible interactions between the different molecules (Are they able to bind to the same receptor(s), and if so, are they acting in a redundant, agonistic, or antagonistic way? Do they share response pathways?).

## Concluding remarks and further perspectives

The fact that indole, cyclo(Phe-Pro), and DPO control virulence factor production in vibrios makes the production and detection mechanisms of these quorum sensing molecules interesting targets for the development of novel drugs to control *Vibrio* disease. A few analogs of these molecules, including the auxins indole-3-acetic acid and indole-3-acetamide and the cyclic dipeptide cyclo(valine-valine), have shown to be promising leads, as they decreased virulence and/or virulence factor production in vibrios [23,46]. In addition to this, a library of synthetic cyclic dipeptides has been screened in *V*. *fischeri*, and one of the compounds showed up to 95% luminescence inhibition [47]. It would be interesting to screen more compounds for increased activity and to establish structure–activity relations with respect to impact on virulence or virulence-related phenotypes. In addition to synthetic compounds with increased activity, natural sources of these quorum sensing molecules could be investigated. Indeed, indoles and cyclic dipeptides are produced by various organisms, including marine bacteria, fungi, and algae [48,49]. Either purified compounds could be applied, or selected (micro)organisms able to produce high levels of these compounds—e.g., lactobacilli or bifidobacteria producing indoles and/or cyclo(Phe-Pro) [50,51]. The application of such compounds to control disease might not be limited to vibrios, because other bacteria, such as *P*. *aeruginosa* and *E*. *coli*, have also been reported to produce and/or respond to these amino acid–derived quorum sensing molecules [35,37,38]. The identification and development of therapeutics derived from these molecules will need to include experiments in appropriate host models because higher organisms can also respond to these amino acid–derived quorum sensing molecules. Indeed, indole has been reported to increase human epithelial cell tight-junction resistance and to reduce inflammatory cytokine levels [52], and several studies suggested a beneficial effect on oxidative stress and inflammation [38]. More recently, cyclo(Phe-Pro) has been documented to induce DNA damage by increasing the levels of reactive oxygen species in mammalian cells [53] and to inhibit innate immune responses [54,55].
